# Swyer‐James‐MacLeod Syndrome Presenting With Hemoptysis Managed by Bronchial Artery Embolization

**DOI:** 10.1002/rcr2.70220

**Published:** 2025-05-28

**Authors:** Dhiran Sivasubramanian, Karthick Balasubramanian, Virushnee Senthilkumar, Sathwik Sanil, Smrti Aravind, Nithish Nanda Palanisamy

**Affiliations:** ^1^ Department of Cardiology Children's Hospital of Philadelphia Philadelphia Pennsylvania USA; ^2^ Department of Critical Care Medicine Christian Medical College Vellore India; ^3^ Department of General Medicine Coimbatore Medical College Coimbatore India; ^4^ Institute of Oncology, Sri Ramakrishna Hospital Coimbatore India

**Keywords:** bronchial artery embolization, haemoptysis, HRCT, Swyer‐James‐MacLeod Syndrome, unilateral hyperlucency

## Abstract

Swyer‐James‐MacLeod syndrome (SJMS), though typically diagnosed in childhood, can present in adults with symptoms like hemoptysis and may be misdiagnosed as other pulmonary conditions like pulmonary embolism. Bronchial artery embolization is an effective treatment for controlling significant hemoptysis in SJMS.

## Graphical abstract

Swyer‐James‐MacLeod syndrome (SJMS) can present in adults with symptoms like hemoptysis and may be misdiagnosed as other pulmonary conditions like pulmonary embolism. Bronchial artery embolization is an effective treatment for controlling significant hemoptysis in SJMS.

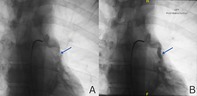

A 29‐year‐old Indian male presented with a year‐long history of hemoptysis, escalating over the past month to 15–20 mL per episode, occurring approximately 10 times daily. He had a history of childhood‐onset asthma, with episodic wheezing and breathlessness triggered by cold and dust exposure. There was no history of tuberculosis or other comorbidities. Physical examination revealed coarse crepitations in the left infrascapular area, while vital signs were normal. Pulmonary function test showed FVC 3.14 L (77.4% predicted), FEV_1_ 2.21 L (62.9%), and FEV1/FVC 0.70. Chest radiography showed a hyperlucent left lung (Figure 1). Computed tomography (CT) revealed diffuse hyperlucent areas, along with fibrotic and bronchiectatic changes in the left lower lobe and lingula, reduced left lung volume, hypoplastic left pulmonary arteries, and a hypertrophied left bronchial artery (Figure 2)—findings consistent with SJMS. Given the persistent and worsening hemoptysis and the presence of a hypertrophied bronchial artery, the patient underwent bronchial artery embolization (Figure 3). Post‐procedure, hemoptysis ceased, and the patient remained stable during follow‐up. SJMS is a rare pulmonary disorder characterised by unilateral hyperlucency, hypoplasia of the pulmonary vasculature, and the presence of bronchiectasis [1, 2]. While often diagnosed in childhood, adult presentations can occur and may mimic other pulmonary conditions [1, 2], leading to misdiagnosis. Bronchial artery embolization can be an effective treatment for significant hemoptysis associated with SJMS.

**FIGURE 1:**
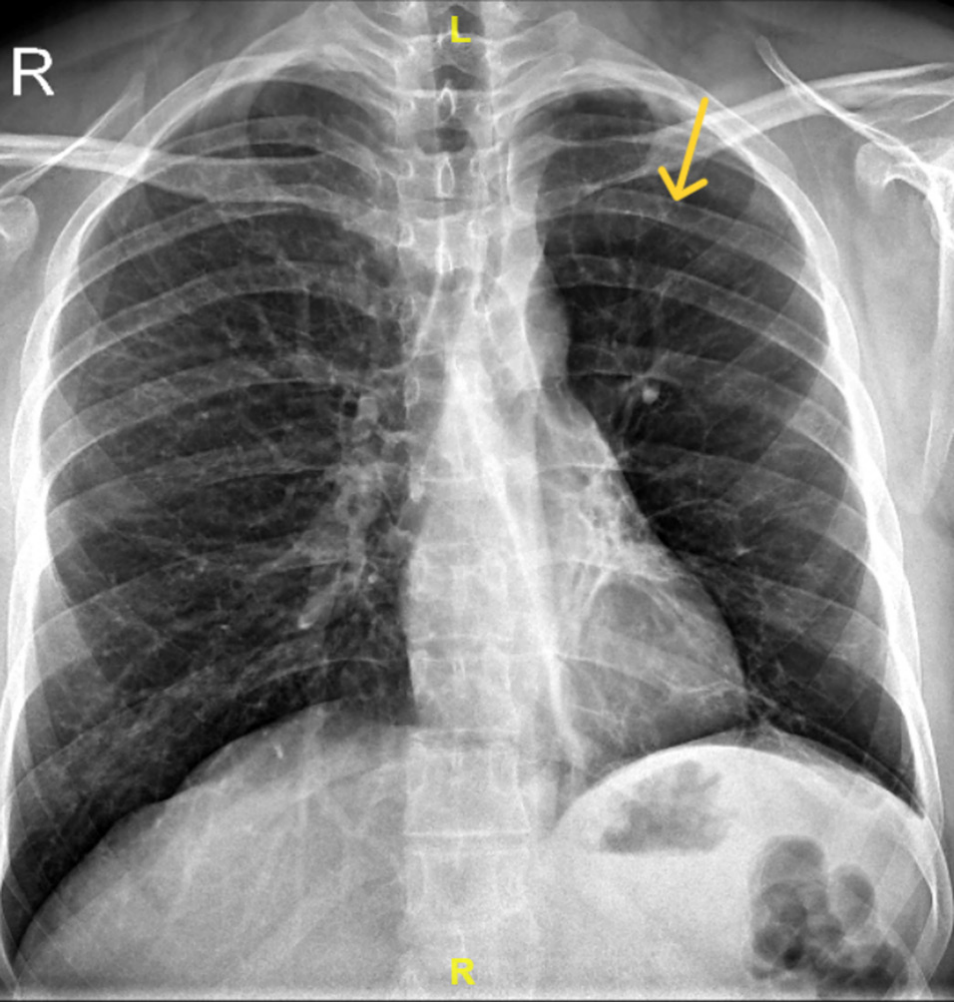
Plain chest radiography (X‐Ray) showing a hyperlucent left lung.

**FIGURE 2:**
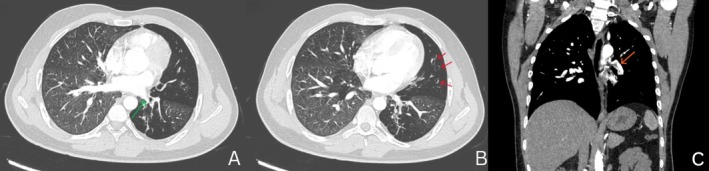
Computed tomography (CT) of the chest (A–C). (A) and (B) axial high‐resolution CT images showing diffuse hyperlucent areas in the left lung, along with fibrotic and bronchiectatic changes (red arrows) in the lower lobe and lingula, reduced left lung volume, and a hypoplastic left pulmonary artery (green arrow)—features suggestive of Swyer‐James‐MacLeod syndrome. (C) Coronal CT pulmonary angiogram image showing hypertrophied left bronchial artery (orange arrow).

**FIGURE 3:**
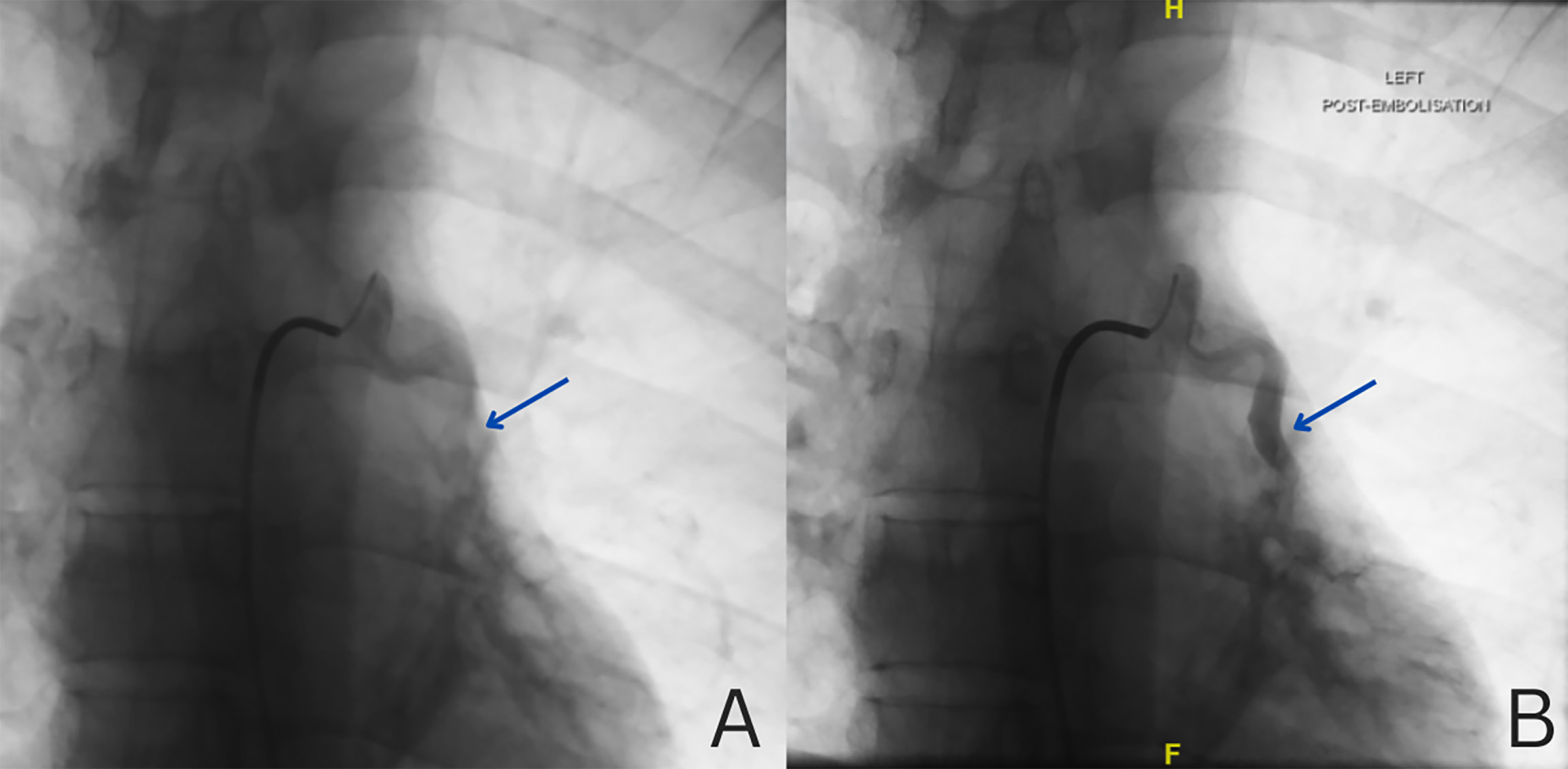
(A) Pre and (B) Post left bronchial artery embolization.

## Author Contributions

**Dhiran Sivasubramanian:** conceptualization, data curation, project administration, supervision, original draft writing, review, and editing. **Karthick Balasubramanian:** patient evaluation, conceptualization, investigation, review, and editing. **Virushnee Senthilkumar:** conceptualization, data curation, investigation, and project administration. **Sathwik Sanil:** conceptualization, investigation, project administration. **Smrti Aravind:** visualization, writing – review, and editing. **Nithish Nanda Palanisamy:** image selection, data curation.

## Ethics Statement

All the data of this study were taken from the medical records of the patient. This report does not contain any personal information that could lead to the identification of the patient.

## Consent

The authors declare that written informed consent was obtained for the publication of this manuscript and accompanying images using the consent form provided by the Journal.

## Conflicts of Interest

The authors declare no conflicts of interest.

## Data Availability

Data sharing is not applicable to this article as no new data were created or analyzed in this study.
